# Single-Cell Transcriptomics and *In Situ* Morphological Analyses Reveal Microglia Heterogeneity Across the Nigrostriatal Pathway

**DOI:** 10.3389/fimmu.2021.639613

**Published:** 2021-03-29

**Authors:** Oihane Uriarte Huarte, Dimitrios Kyriakis, Tony Heurtaux, Yolanda Pires-Afonso, Kamil Grzyb, Rashi Halder, Manuel Buttini, Alexander Skupin, Michel Mittelbronn, Alessandro Michelucci

**Affiliations:** ^1^ Luxembourg Centre for Systems Biomedicine (LCSB), University of Luxembourg, Esch-sur-Alzette, Luxembourg; ^2^ Luxembourg Center of Neuropathology (LCNP), Luxembourg, Luxembourg; ^3^ Department of Life Sciences and Medicine (DLSM), University of Luxembourg, Esch-sur-Alzette, Luxembourg; ^4^ Neuro-Immunology Group, Department of Oncology (DONC), Luxembourg Institute of Health (LIH), Luxembourg, Luxembourg; ^5^ Faculty of Science, Technology and Medicine, University of Luxembourg, Belvaux, Luxembourg; ^6^ National Center for Microscopy and Imaging Research, University of California San Diego, La Jolla, CA, United States; ^7^ Department of Oncology (DONC), Luxembourg Institute of Health (LIH), Luxembourg, Luxembourg; ^8^ National Center of Pathology (NCP), Laboratoire National de Santé (LNS), Dudelange, Luxembourg

**Keywords:** microglia, nigrostriatal pathway, single-cell transcriptomics, cellular heterogeneity, immune alerted, cell morphology, Parkinson’s disease

## Abstract

Microglia are the resident immune effector cells of the central nervous system (CNS) rapidly reacting to various pathological stimuli to maintain CNS homeostasis. However, microglial reactions in the CNS may also worsen neurological disorders. Hence, the phenotypic analysis of microglia in healthy tissue may identify specific poised subsets ultimately supporting or harming the neuronal network. This is all the more important for the understanding of CNS disorders exhibiting regional-specific and cellular pathological hallmarks, such as many neurodegenerative disorders, including Parkinson’s disease (PD). In this context, we aimed to address the heterogeneity of microglial cells in susceptible brain regions for PD, such as the nigrostriatal pathway. Here, we combined single-cell RNA-sequencing with immunofluorescence analyses of the murine nigrostriatal pathway, the most affected brain region in PD. We uncovered a microglia subset, mainly present in the midbrain, displaying an intrinsic transcriptional immune alerted signature sharing features of inflammation-induced microglia. Further, an *in situ* morphological screening of inferred cellular diversity showed a decreased microglia complexity in the midbrain when compared to striatum. Our study provides a resource for the identification of specific microglia phenotypes within the nigrostriatal pathway, which may be relevant in PD.

## Introduction

Microglia are the resident immune effectors of the brain that arise from the yolk sac and colonize the brain early during embryonic development (1). Under homeostatic conditions, both during development and in the adult brain, microglia play key roles shaping the neuronal network through synaptic pruning and phagocytosis of apoptotic neurons (2–5). In addition, microglia scan the adult brain and can rapidly react to threatening conditions to mainly maintain the brain homeostasis. In this context, improper immune responses, such as weakened or exaggerated microglia reactions, can play a critical role in the development and progression of neurological diseases with an immunological component (6–8). Thus, specific microglial poised subsets ultimately supporting or harming the neuronal network may contribute to the development and progression of CNS disorders exhibiting regional-specific and cellular pathological hallmarks, such as many neurodegenerative disorders, including Parkinson’s disease (PD).

PD is the second most common neurodegenerative disease of aging and the most frequent movement disorder (9). The presence of intracellular inclusions of misfolded alpha-synuclein (α-syn) and the loss of dopaminergic neurons in the substantia nigra pars compacta (SNc), a basal ganglia structure located in the midbrain, characterize the brain of PD patients. In the healthy brain, the dopaminergic neurons in the SNc mainly support the basal ganglia circuit by supplying the striatum with dopamine. Consequently, dopamine levels in the dorsal striatum of PD patients are decreased, triggering the impairment of the nigrostriatal pathway leading to various non-motor and classical motor dysfunctions, including bradykinesia, tremor, posture impairment or rigidity (10–12). Importantly, α-syn aggregation and loss of dopaminergic neurons are associated with neuroinflammation, which also constitutes a hallmark of PD (13). However, the effective role of the neuroinflammatory processes in PD is still unclear. For example, it has not yet been elucidated if specific microglia subsets within the nigrostriatal pathway may play a causative or a protective role for the development and progression of PD (14–16).

In the recent years, microglia heterogeneity in the healthy brain is emerging (17–19). Specifically, microglia diversity across various brain regions has been described at the level of their density, morphology and transcriptional programs (20–22). Further, single-cell RNA-sequencing studies enabled to detect microglia heterogeneity beyond region specificity, unraveling specific microglia subsets within different brain regions (23). Still, none of the previous studies has addressed the heterogeneity of microglial cells in susceptible brain regions for PD, such as the nigrostriatal pathway, at single-cell resolution.

Here, we conducted single-cell RNA-sequencing of the midbrain and striatum in 6-month-old female mice and identified specific microglia subsets characterized by different immune programs. Among them, we detected a subset, mainly composed by microglial cells isolated from the midbrain, displaying an intrinsic immune alerted state sharing genes characterizing microglia under inflammatory conditions (24). Further, we combined single-cell RNA-sequencing analyses with morphological and protein screening of inferred cellular diversity taking cortical microglia as a paradigm for the homeostatic resting state and cerebellar microglia as cells displaying an immune alerted state (21). In line with the single-cell transcriptomics results and their typical resting state, microglia from the cortex and striatum showed higher ramification length and increased branching points compared to microglia from the cerebellum and midbrain. Lastly, we found heterogeneity of microglial cell density within midbrain sub-regions, with the number of Iba1+ cells being higher in the substantia nigra pars reticulata (SNr), similarly to cortex and striatum, while being lower in the SNc and ventral tegmental area (VTA), comparable to cerebellum.

Taken together, our results shed light on the complexity of microglial cell diversity in the nigrostriatal pathway and establish a resource for the identification of specific phenotypes, which might be relevant for the development of PD.

## Materials And Methods

### Animals

C57BL/6J female mice were housed in individually ventilated cages (IVC) in a conventional animal facility at the University of Luxembourg in agreement to the EU Directive 2010/63/EU and Commission recommendation 2007/526/EC. Mice were kept in groups under a dark-light cycle with *ad libitum* access to water and food. The animal work of the present study has been conducted and reported in accordance with the ARRIVE (Animal Research: Reporting of In Vivo Experiments) guidelines to improve the design, analysis and reporting of research using animals, maximizing information published and minimizing unnecessary studies.

### Microglia Isolation, RNA Extraction and RT-PCR

Six-month-old C57BL/6J female mice were euthanized in deep anesthesia (intraperitoneal injection of medetomidine 1 mg/kg and ketamine 100 mg/kg) and perfused with PBS. Mouse brains were manually dissected into cortex, cerebellum, midbrain and striatum and tissue was dissociated with Adult Brain Dissociation Kit (Miltenyi Biotec). Microglia were subsequently isolated by magnetic separation. Briefly, a total of 1x10^7^ cells was incubated with 90 µl of MACS buffer and 10 µl of CD11b microbeads antibody (Milteny Biotec) for 20 min at 4°C. Total RNA was extracted from eluted microglial cells using NucleoSpin RNA Plus XS (Macherey-Nagel) for samples containing less than 100.000 CD11b+ cells, whereas the kit innuPREP RNA Mini Kit 2.0 (Analytik Jena) was used for samples constituted by more than 100.000 CD11b+ cells. We measured the RNA concentration and quality by Nanodrop (Nanodrop technologies) and we performed reverse transcription using the ImProm-II reverse Transcription System (Promega) according to manufacturer’s instructions. Reverse transcription was performed at 25°C for 5 min, followed by 42°C for 60 min and 70°C for 15 min. For the RT-PCR conducted in 96-well plates, 2 µl of cDNA were mixed with 10 µl of iQ SYBR Green Supermix (Biorad) and 0.5 µl of 10 µM primers. A total volume of 20 µl was added to a LigthCycler 480 Multiwell Plate 96 white (Roche) and RT-PCR was performed in the LigthCycler 480 II (Roche) using the following program: 95°C for 3 min, 40 cycles at 95°C for 3 sec, 60°C for 3 sec and 72°C for 3 sec. For the RT-PCR conducted in 384-well plates, 1 µl of cDNA was mixed with 2.5 µl of SYBR Green Mastermix (Applied Biosystems) with 1.25 µl of water and 0.125 µl of 10 µM primers. A total volume of 5 µl was added to a MicroAmp Optical 384 well-reaction plate (Applied Biosystems) and RT-PCR was performed in the QuantStudio Design & Analysis software (Applied Biosystems) using the following program: 95°C for 3 min, 40 cycles at 95°C for 3 sec, 60°C for 3 sec and 72°C for 3 sec. The primer sequences were as follows. *Gapdh* forward: TGCGACTTCAACAGCAACTC, *Gapdh* reverse: CTTGCTCAGTGTCCTTGCTG; *Cx3cr1* forward: CCTGCCCTTGCTTATCAT, *Cx3cr1* reverse: GCCTTCTTGCGATTCTTG; *Fcrls* forward: TTCTGGTCTTCGCTCCTGTC, *Fcrls* reverse: ACCGCGTCTTGCATTCCTAA; *P2ry12* forward: GTGCAAGGGGTGGCATCTA, *P2ry12* reverse: TGGAACTTGCAGACTGGCAT. The threshold cycle of each gene was determined as PCR cycles at which an increase in reporter fluorescence above a baseline signal was measured. The difference in threshold cycles between the target gene and reference gene *Gapdh* yielded the standardized expression level (dC_T_). The expression level of each gene was calculated with the formula 2^-dCT^. Data are represented as mean ± standard error of the mean (SEM) from three independent experiments. All statistical analyses were performed using GraphPad Prism 8.0 (GraphPad Software, Inc., San Diego, CA). The significance was analyzed by a one-way ANOVA followed by a post-hoc Tukey’s test. Differences between groups were considered as significant when *p* values were less than 0.05 (* p<0.05, ** p< 0.01, *** p<0.005).

### Single-Cell RNA-Sequencing

#### Tissue Dissection and Library Preparation

Six-month-old C57BL/6J female mice were euthanized in deep anesthesia by intraperitoneal injection of medetomidine (1 mg/kg) and ketamine (100 mg/kg) and perfused transcardially with phosphate-buffered saline (PBS). We manually dissected and isolated striatum and midbrain from mouse brains on ice. We separately dissected those brain regions using Adult Brain Dissociation Kit (Miltenyi Biotec). Cells were re-suspended in 0.5% BSA and the single cell RNA libraries were captured using the Drop-seq method (24). The 3’end enriched cDNA libraries were prepared by tagmentation reaction of 600 pg cDNA sample library using the standard Nextera XT tagmentation kit (Illumina). Reactions were performed according to the manufacturer’s instructions. The PCR amplification cycling program used was 95°C for 30 sec, and twelve cycles at 95°C for 10 sec, 55°C for 30 sec and 72°C for 30 sec, followed by a final extension step at 72°C for 5 min. Libraries were purified twice to reduce primers and short DNA fragments with 0.6× and 1× Agencourt AMPure XP beads (Beckman Coulter), respectively. Lastly, purified libraries were eluted in 10 μl Molecular Grade Water. Quality and quantity of the tagmented cDNA library were evaluated using Bioanalyzer High Sensitivity DNA Chip. The average size of the tagmented libraries before sequencing was between 400 and 700 bps.

Purified Drop-Seq cDNA libraries were pulled together and sequenced using Illumina NextSeq 500 with the recommended sequencing protocol except for 6 pM of custom primer (GCCTGTCCGCGGAAGCAGTGGTATCAACG CAGAGTAC) applied for priming of read 1. Paired-end sequencing was performed for the read 1 of 20 bases (covering the random cell barcode 1–12 bases and the rest 13–20 bases of random unique molecular identifier) and for read 2 of 60 bases of the mRNAs. Raw reads were further de-multiplexed and processed using the Drop-seq computational pipeline (24).

#### Single-Cell RNA-Sequencing Data Analysis, Reads Filtering, Alignment and Mapping Quality

We adopted the Drop-seq method as previously described (24). The identification of the low quality cells was done separately in each data set. In order to select only the highest quality data, we sorted the cells by the cumulative gene expression. A subset of cells with the highest cumulative expression was considered for the analysis (25). Additional to this filtering, we defined cells as low-quality, based on three criteria for each cell. The number of expressed genes is higher than 200 with 2 median-absolute-deviations (MADs) above the median, the total number of counts is 2 MADs above or below the median and the percentage of counts to mitochondrial genes is 1.5 MADs above the median. Cells failing at least two criteria were considered as low quality cells and filtered out from further analysis. Similar to the cell filtering, we filtered out the low abundant genes being expressed in less than 5 cells in the data. Moreover, we excluded the mitochondrial and ribosomal genes from the rest of the analysis. The integration of the filtered matrices of the different tissues was performed using Seurat v3.1 (26). The final gene expression matrix, which we used for downstream analyses, consisted of 1,337 cells and 15,446 genes. The filtered count matrix was normalized for library size per cell, whereby the expression level of each gene was divided by the cell’s total library size, multiplying this by a scale factor (10,000 default), and natural-log transformed the result, using log1p. Principal component analysis (PCA) was computed using the 5000 most variable genes on the aligned data. The clustering of data was performed using Louvain clustering. The resolution of the clustering was selected based on the best silhouette score of the different resolutions (27). The clusters were identified using the graph-based clustering algorithm implemented in Seurat. Then, differential expression analysis was used to identify whether this clustering segregated the expected cell types in the brain. A short list of manually curated markers was used to infer the identity of the different clusters. Cells assigned as “microglia” were re-projected in two dimensions using again the 5,000 most variable genes of this subset. Next, we performed differential expression analysis on the clusters of these projected microglia populations. For this, we used the function FindAllMarkers on the normalized counts using MAST (28) as test with the total number of transcripts in each cell as a covariate (LogFC threshold = 0, min pct = 0) and the Bonferroni correction to correct for multiple hypothesis testing (Padj). From this differential gene expression analysis, one small population was annotated as oligodendrocytes. This cluster was filtered out for further analysis. A new PCA was performed using the normalized count matrix of the remaining “purified microglia” population. The new clusters were checked for difference in gene expression levels. We performed differential expression analysis using a generalized linear method with linear predictors adding as covariate the total number of transcripts in each cell using Monocle2 (29). The Benjamini-Hochberg correction was applied to correct for multiple hypothesis testing (here q values were used as more advanced adjusted p-values, which not only consider the sample size, but also take into account an optimized FDR).

### Flow Cytometry Analyses

Single-cell suspensions were obtained as previously described for single-cell RNA-seq analyses. Cells were re-suspended in ice-cold HBSS with 2% FBS and 10 mM HEPES (FACS buffer) and filtered through a 70 µm nylon mesh (CellTrics). For multicolor phenotyping, cells were blocked with Fc receptor binding inhibitor (anti-mouse CD16/CD32 monoclonal antibody; 1:100; eBioscience) for 15 min at 4°C to reduce binding of non-specific Fc-gamma receptors, and then stained with fluorochrome-conjugated antibodies (anti-mouse CD45-FITC antibody; 1:1000; eBioscience; anti-mouse CD11b-PERCP_Cy5.5; 1:20; eBioscience; anti-mouse CD83-PE; 1:500; Biolegend; anti-mouse CD206-APC; 1:50; Biolegend) for 30 min at 4°C in the dark. Unstained (control) and stained cells were washed and re-suspended in 100 µL of FACS buffer prior acquisition. Before acquisition, the performance of the instrument was assessed using CS&T beads according to the manufacturer’s instructions. Single-stain controls were prepared with UltraComp eBeads (eBioscience) following the manufacturer’s instructions and thus used to calculate the compensation matrix. Hoechst (0.1 µg/ml, Bisbenzimide, 33342; Sigma) was added for dead cell discrimination. Samples were run on FACSAria IIu SORP cytometer (Becton Dickinson) and flow cytometry data were analyzed using FlowJo software (v. 10.6.1, Becton Dickinson).

### Immunohistochemistry Analyses

Brains were fixed in 4% PFA for 24h and kept in PBS with 0.1% NaN_3_. They were cut with vibratome (VT1000S from Leica) into 50 µm sagittal free-floating sections and kept at -20°C in a cryoprotective medium (1:1 v/v PBS/Ethylene Glycol, 10g.L^-1^ Polyvinyl Pyrrolidone). For immunohistochemistry, sections were washed (PBS), permeabilized (PBS with 3% H_2_O_2_ and 1.5% Triton X-100) and blocked (PBS with 5% BSA). Sections were then incubated overnight (PBS with 0.3% Triton X-100 and 2% BSA) with IBA1 (1:1000; Wako) and tyrosine hydroxylase (TH; 1:1000; ab76442) antibodies. IBA1 was visualized using donkey anti-rabbit IgG Molecular Probes Alexa Fluor 647, while TH using goat anti-chicken IgG Molecular Probes Alexa Fluor 488 (Thermo Fisher). Sections were mounted on glass slides using DAPI-Fluoromount-G (SouthernBiotech). Sections were imaged at 20x using Zeiss Confocal LSM-710. For microglial morphological analyses, Z-stack pictures were taken and at least 12 cells per region in four mice were analyzed using IMARIS software (Bitplane). Lastly, we used GraphPad Prism 8 software for statistical analyses. For parametric groups (cell density and process length), we applied one-way ANOVA with post-hoc Tukey’s test. We analyzed non-parametric groups (number of branching points and number of segments) by using Kruskal-Wallis followed by post-hoc Dunn’s test.

## Results

### Single-Cell Transcriptomics Identifies Cellular Diversity of the Midbrain and Striatum

**Figure 1:**
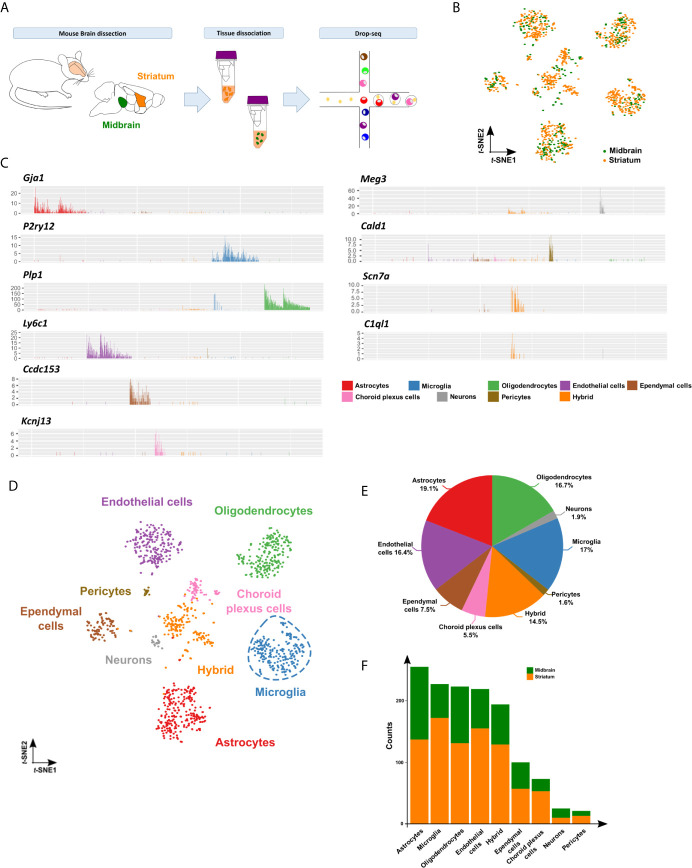
Single-cell transcriptomics identifies cellular diversity of the midbrain and striatum. **(A)** Schematic representation of the experimental approach. Tissue dissected and dissociated from midbrain (in green) and striatum (in orange) of six months-old C57BL/6J mice (pool of 5 mice) analyzed by Drop-seq. **(B)** *t*-SNE projection of 1,337 single cells included in the study showing 480 cells isolated from midbrain (in green) and 857 cells from striatum (in orange). **(C)** Bar plots of representative cell type-specific markers across nine identified clusters: microglia (*P2ry12*), astrocytes (*Gja1*), oligodendrocytes (P*lp1*), endothelial cells (L*y6c1*), hybrid (*Scn7a* and *C1ql1)*, ependymal cells (*Ccdc153*), choroid plexus cells (*Kcnj13*), neurons/neuronal stem cells (*Meg3* and *Snhg11*) and pericytes (*Cald1*, *Vtn*, *Notch3*) clusters. See **Figure S1** and **Table S1** for additional cell type-specific markers used for cluster annotation. **(D)** Annotated clusters in the *t*-SNE map showing nine specific groups identified by differential expression analysis featuring 15,446 total genes (FDR<0.05): microglia (in blue), astrocytes (in red), oligodendrocytes (in green), endothelial cells (in purple), hybrid (orange), ependymal cells (brown), choroid plexus cells (in pink), neurons/neuronal stem cells (in grey) and pericytes (in pale brown). **(E)** Pie chart depicting the percentage of the identified cell types. **(F)** Bar plot showing proportion of cell types within midbrain (in green) and striatum (in orange).

To analyze the cellular and molecular heterogeneity of the nigrostriatal pathway at single-cell resolution, we manually dissected the midbrain and striatum from five 6 month-old C57Bl/6J female mice and used the microfluidic Drop-seq method for single-cell transcriptomics analyses (30) **(** **Figure 1A** **)**. Unsupervised-clustering and t-distributed stochastic neighbor-embedding (*t*-SNE) projection represented single cells separated into individual clusters. Among 1,337 cells considered for subsequent analyses, 480 cells were from the midbrain and 857 cells from the striatum (**Figure 1B**). Differential expression analysis featuring 15,446 most variable genes between the clusters (FDR<0.05) identified nine specific groups. To gather the resultant identity of the clusters, we analyzed the expression levels of the top genes in each cluster (**Table S1**). We annotated them based on cell type-specific gene markers (31, 32). Specifically, we identified four main clusters, corresponding respectively to astrocytes (e.g. *Gja1*, *Plpp3*, *Slc1a2*, *Aqp4*), microglia (e.g. *P2ry12*, *Hexb*, *Cx3cr1*, *Siglech*), oligodendrocytes (e.g. *Plp1*, *Mbp*, *Mobp*, *Trf*) and endothelial cells (e.g. *Ly6c1*, *Cldn5*, *Pltp*, *Pecam1*). Four smaller clusters were represented by ependymal cells (e.g. *Ccdc153*, *Tmem212*, *Dynlrb2*, *Rsph4a*), choroid plexus cells (*Kcnj13*, *Flor1*, *Clic6*, *Kl*) mainly constituted by striatal cells, neurons/neural stem cells (*Meg3*, *Snhg11*, *Ndrg4*, *Snap25*) and pericytes (e.g. *Cald1*, *Vtn, Notch3, Snap25*). Lastly, we identified a hybrid cluster represented by a mix of cell-types, including cells expressing neuronal (*Scn7a*, *Map2*) or oligodendrocyte precursor cell (*C1ql1*, *Pdgfra*) markers (**Figure 1C**; **Figure S1**). Next, following the comprehensive characterization of the clusters (**Figure 1D**), we verified that identities, markers, and proportions of cell types matched previous single-cell droplet-based sequencing data from mouse brain tissue (**Figure 1E**) (33), indicating that our results were robust for analyses. Lastly, we showed that cell type distribution was similar across midbrain and striatum, confirming that both cell suspensions contained the brain cell types described above (**Figure 1F**).

Overall, our single-cell approach enabled to identify in an unbiased manner different cell types present in the nigrostriatal pathway, allowing studying them separately and at single-cell resolution.

### Microglia Within the Nigrostriatal Pathway Segregate Into Specific Immune Subsets

**Figure 2:**
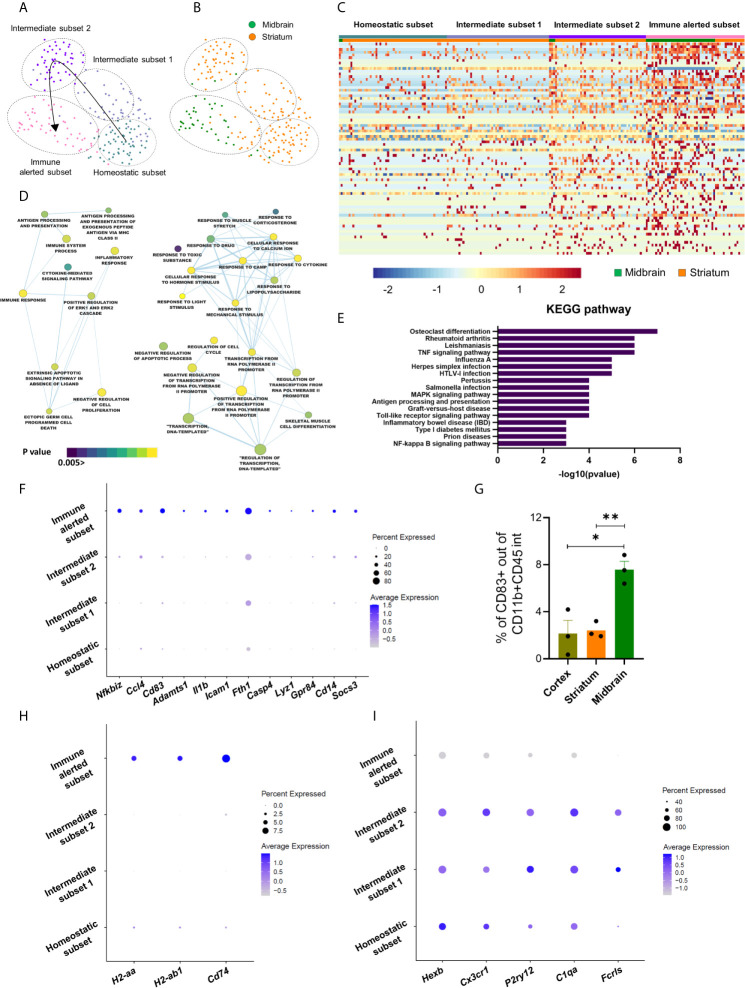
Microglia within the nigrostriatal pathway segregate into specific immune subsets. **(A)** UMAP plot of the re-projected microglia cluster showing four distinct subsets: homeostatic (in grey), intermediate 1 (in pale blue), intermediate 2 (in purple) and immune alerted (in pink). **(B)** UMAP representation showing 210 microglial cells, with 41 cells from the midbrain (in green) and 169 cells from striatum (in orange). **(C)** Heatmap showing clustering analysis of single cells, featuring 78 differential expressed genes across the four subsets (q value < 0.05). Color bar represents z-scores (from low z-score in blue to high z-score in red) (**Table S2**). **(D)** Cytoscape network analysis of gene ontology terms identified by DAVID analysis (p value < 0.05, node cut off q value < 0.1) of the 78 differentially expressed genes across microglia subsets (**Table S3**). **(E)** Top 17 KEGG pathways identified by DAVID resulting from 78 differentially expressed genes (**Table S3**). **(F)** Dot plot representing the expression of inflammatory genes across the microglia subsets. Circle diameter denotes percent expression; color code indicates average expression levels. **(G)** Percentage of CD83+ cells within the CD11b+CD45int population quantified by flow cytometry. Bars represent mean ± SEM (cortex in pale green; striatum in orange; midbrain in green). Unpaired Student t test (n = 3) (*p < 0.05, **p < 0.01). **(H–I)** Dot plots representing **(H)** antigen presenting cell markers and **(I)** homeostatic genes across the microglia subsets. Circle diameter denotes percent expression; color code indicates average expression levels.

As we were interested to elucidate the heterogeneity of microglial cells, we selected the corresponding cluster for downstream analyses. First, by testing the purity of the microglia-associated cluster, we identified few cells expressing oligodendrocytic markers, such as *Mbp*, *Mag* or *Plp1*, thus we discarded these cells for subsequent analyses (**Figure S2**). Uniform Manifold Approximation and Projection (UMAP) representation of 210 uncontaminated microglial cells, with 169 cells harvested from the striatum and 41 cells from the midbrain, revealed four different subsets, namely homeostatic, intermediate 1 and 2 and immune alerted (**Figure 2A**). They all contained striatal microglial cells, with a higher proportion of cells in the homeostatic and intermediate subsets, while microglia from the midbrain mostly clustered to the immune alerted subset (**Figure 2B** **).** To detect transcriptional differences between the four subsets, we first performed differential expression analysis and identified 78 genes (q value < 0.05) (**Figure 2C**; **Table S2**). Corresponding gene ontology analysis using DAVID (34, 35) revealed biological processes associated to “inflammatory response”, “cytokine-mediated signaling pathway”, “antigen processing and presentation” and “response to lipopolysaccharide” (**Figure 2D**). In line with these terms, KEGG analysis revealed pathways underlying microglia activation, such as TNF, MAPK, Toll-like receptor and NFκB signaling pathways (**Figure 2E**; **Table S3**). Indeed, among the differentially expressed genes, inflammatory markers, such as *Nfkbiz, Ccl4, Cd83, Adamts1, Il1b, Icam1, Fth1*, *Casp4*, *Lyz1, Gpr84*, *Cd14*, *Socs3* were up-regulated in the immune alerted subset (**Figure 2F**). We confirmed increased amounts of CD83+ cells among CD11b+CD45int cells, representing microglia, in the midbrain (7.58 ± 0.7%) compared to cortex (2.15 ± 1.1%) and striatum (2.42 ± 0.4%) by flow cytometry analyses (**Figure 2G**; **Figure S3**). Additionally, antigen presenting cell markers, including *H2-aa*, *H2-ab1* or *Cd74* were specifically up-regulated in the immune alerted subset (**Figure 2H**), while this subset expressed lower levels of the microglia homeostatic genes, such as *Hexb, Cx3cr1*, *P2ry12, C1qa* and *Fcrls* (**Figure 2I**). This is reminiscent of the decrease of homeostatic genes in reactive microglia under inflammatory conditions, suggesting that cells belonging to this cluster display an immune alerted-like state. We considered the two intermediate subsets as transitions between homeostatic and immune alerted microglia (**Figure 2A**). To exclude that the immune alerted subset could be, at least partially, constituted by non-parenchymal macrophages that mediate immune responses at brain boundaries, namely border-associated macrophages (BAMs), we verified the expression levels of their recently described specific markers (36–40). Except for *Cd74* and *H2-Ab1*, which are known genes to be also expressed by activated microglia (41), additional specific BAM markers were not detected (e.g. *Lyve1* and *Ccr2*) or expressed by few cells not pertaining to the immune alerted cluster (e.g. *Mrc1*) (**Figure S3**), thus indicating that the identified microglia subsets were mainly constituted by microglial cells. Flow cytometry analyses aimed at examining the expression of CD206 (encoded by *Mrc1*) in CD11b+CD45+ cells (approximately 2-3%) did not detect differences across the analyzed brain regions, hence showing that the midbrain is not enriched with BAMs when compared to striatum and cortex (**Figure S3**).

A recent study described microglia in the cerebellum to be more immune vigilant than cortical microglia (21). Therefore, we examined the expression of microglia homeostatic genes in freshly isolated microglia from midbrain or striatum and compared them with microglia harvested from cortex and cerebellum. For this, we manually dissected the cortex, cerebellum, striatum and midbrain from 6-month C57Bl/6J mouse females and extracted RNA from MACS-isolated CD11b+ cells. In agreement with previous observations (21), microglia isolated from the cerebellum expressed lower levels of the homeostatic genes (e.g. *Cx3cr1, Fcrls, P2ry12)* when compared to the cortex. In line with the results obtained at single-cell resolution, we detected a trend to a decrease of the homeostatic genes in the midbrain compared to the striatum, although these differences did not reach statistical significance (**Figure S3**), probably due to the heterogeneity that we detected within these brain regions at single-cell resolution.

Lastly, besides differences in their immune phenotype, microglial cells in the immune alerted subset exhibited an up-regulation of genes related to TGF-β signaling (*Atf3*, *Egr1*), epigenetic functions (*H3f3b*, *Chd4*), proliferation (*Btg2*), reactive oxygen species-mediated cell death (*Gpx1*), energy production (*Atp5a1*) and autophagy-linked genes (*Vps29*) (**Table S2**).

Taken together, our data suggests that microglial cells in the striatum and midbrain display different immune phenotypes, with the latter enriched by immune alerted microglia. Further, bulk analyses strengthen the importance of single-cell transcriptomics studies, which enabled to detect unprecedented transcriptional microglia subsets across the nigrostriatal pathway.

### Midbrain-Enriched Immune Alerted Microglia Show Transcriptional Similarities to Inflammation-Associated Microglia and May Be Supported by Other CNS Glial Cells

**Figure 3:**
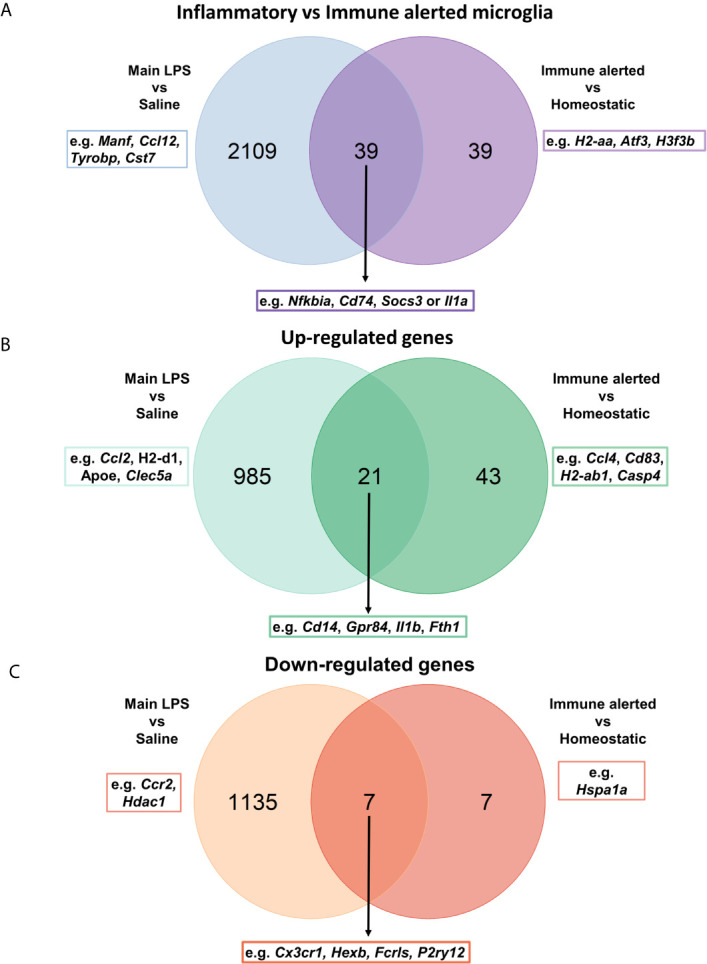
Midbrain-enriched immune alerted microglia show transcriptional similarities to inflammation-associated microglia. **(A–C)** Venn diagrams showing the comparison between 78 differentially expressed genes across midbrain/striatum microglia subsets and 2,148 genes characterizing inflammation-associated microglia (24).

Next, to further characterize the immune transcriptional profile of midbrain-enriched immune alerted microglia, we compared its signature with microglia under acute systemic inflammatory conditions gathering the corresponding dataset from our recent study conducted at single-cell resolution using the Drop-seq technology (24). Intriguingly, our previous gene ontology analysis detected biological processes related to “response to lipopolysaccharide”. In this context, the comparison of midbrain-enriched immune alerted microglia signature with microglia from lipopolysaccharide-injected mice identified 50% (39 out of 78) of shared differential expressed genes (e.g. *Nfkbia*, *Cd74*, *Socs3* or *Il1a*) (**Figure 3A**). Among these genes, 72% (28 out of 39) were up- or down-regulated in both immune alerted and inflammation-associated microglia populations. Specifically, genes such as *Cd14*, *Gpr84*, *Il1b* and *Fth1* were upregulated in both populations, while *Ccl4*, *Cd83*, *H2-ab1*, *Casp4* were exclusively overexpressed in the immune alerted subset (**Figure 3B**). Further, genes like *Cx3cr1, Hexb, Fcrls* and *P2ry12* were downregulated in both microglia populations, whereas *Hspa1a* was solely decreased in the midbrain-enriched microglia population (**Figure 3C**).

In the CNS, the intense crosstalk of microglial cells with neurons and the other glial cells, including astrocytes, oligodendrocytes and ependymal cells, is critical for the acquisition of the microglial phenotype. Hence, we sought to analyze differentially expressed genes between midbrain and striatum within the corresponding cell clusters (**Table S4**). The low number of neurons isolated with our protocol did not enable us to analyze their differentially expressed genes between the two regions. In contrast, we detected differentially expressed genes across the other glial cells, especially in astrocytes and ependymal cells. When focusing on highly up-regulated genes (logFC > 1; adj p value < 0.05), the expression levels of *Vwa1, Xpr1, H2-T3* and *Foxb1* in astrocytes were increased in the midbrain compared to striatum. Interestingly, *H2-T3* gene, coding for H-2 class I histocompatibility antigen, has been shown to be up-regulated in murine astrocytes under IFNγ exposure (42). On a side note, supporting the accuracy of our dissected brain regions, high expression levels of the transcription factor *Foxb1* (forkhead box B1) in the midbrain are in line with its specific expression in diencephalic brain regions, such as the *substantia nigra* (43). In this perspective, the *Ttr* gene coding for transthyretin, a protein that in the brain is mainly produced in the choroid plexus (44), was overexpressed in all the main identified cell types of the striatum (**Table S4**). In ependymal cells, the expression levels of *Car9, Fam81b, Atp5f1, Sparcl1, Cfap36* and *Fos* genes were up-regulated in the midbrain. Of note, *Atp5f1* gene encodes a subunit of mitochondrial ATP synthase, the enzyme that catalyzes the production of ATP. Microglia are able to sense and catabolize extracellular ATP, which triggers the recruitment of microglial protrusions and is converted into AMP and adenosine (45).

Taken together, we show that midbrain-enriched immune alerted microglia share transcriptional features of inflammatory and reactive microglia. Transcriptional differences of other glial cells may support the identified “immune alerted” phenotype of microglia in the midbrain.

### Microglial Density and Morphology Across Midbrain and Striatum Are Heterogeneous

**Figure 4:**
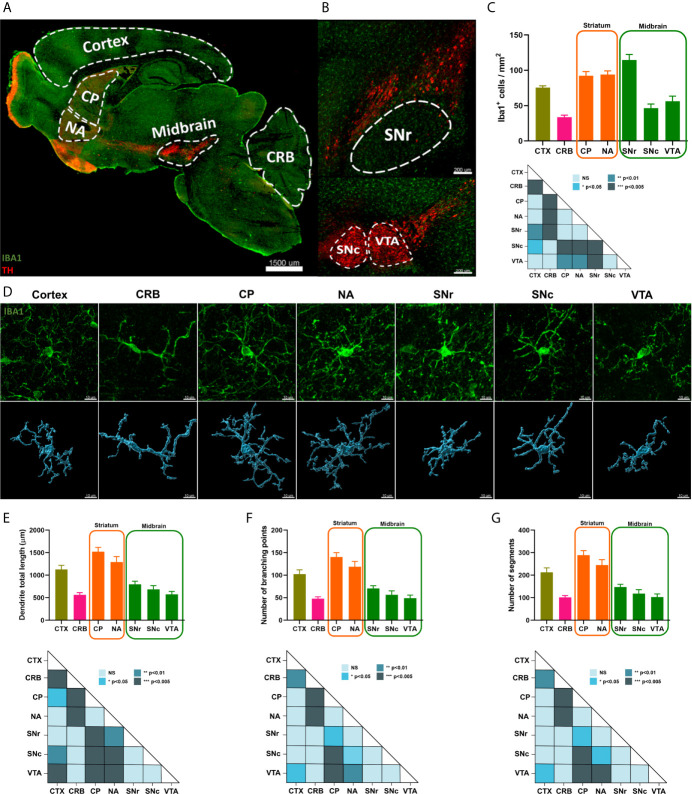
Microglial density and morphology across striatum and midbrain are heterogeneous. **(A)** Representation of different areas analyzed for microglia density and morphology. TH was employed to identify the brain regions, while IBA1 was used to visualize microglia. Scale bar, 1500 µm (CP, caudoputamen; NA, nucleus accumbens; CRB, cerebellum). **(B)** Tile pictures showing midbrain sub-regions in sagittal mouse brain. Scale bar, 200 µm (SNr, substantia nigra pars reticulata; SNc; substantia nigra pars compacta; VTA, ventral tegmental area). **(C)** Quantification of microglial cell density in the different brain areas, cortex (in pale green), cerebellum (in pink), striatum (in orange) and midbrain (in green). Bars represent the mean ± standard error of the mean (SEM) from four independent mice. One-way ANOVA with post-hoc Tukey’s test was used for statistical test (*p < 0.05, **p < 0.01, ***p < 0.005). **(D)** 3D reconstruction of representative microglial cells across different brain regions. Scale bar, 10 μm (CRB, cerebellum; CP caudoputamen; NA, nucleus accumbens; SNr, substantia nigra pars reticulata; SNc, substantia nigra pars compacta; VTA, ventral tegmental area). **(E-G)** Quantification of microglial cell complexity across brain regions. Bars represent the mean ± standard error of the mean (SEM) from twelve independent Iba1+ cells. One-way ANOVA with post-hoc Tukey’s test was used for statistical test for process total length, whereas Kruskal-Wallis followed by post-hoc Dunn’s test was used as statistical test for number of branching points and number of segments (NS not significant, *p < 0.05, **p < 0.01, ***p < 0.005) (**Table S5**). CTX, cortex; CRB, cerebellum; CP caudoputamen; NA, nucleus accumbens; SNr, substantia nigra pars reticulata; SNc, substantia nigra pars compacta; VTA, ventral tegmental area.

Then, we studied microglial cell density and morphology across the previously analyzed brain regions. Anatomically, two main subregions, namely the caudoputamen (CP) and the nucleus accumbens (NA), constitute the mouse striatum (**Figure 4A**), whereas three subregions, the SNc, SNr and VTA, compose the midbrain (**Figure 4B**). To link morphology to functionality, we also included cortex and cerebellum in our analyses since microglia from these two brain regions have been previously described to have different immunological phenotypes (21). We used IBA1 antibody to study microglia and tyrosine hydroxylase (TH) to localize the different brain regions and subregions in the mouse tissue by immunofluorescence analyses. We identified different patterns of microglial cell density, with CP, NA and SNr subregions composed by similar shapes than cortex, whereas microglia density in the SNc and VTA was closer to cerebellum. Notably, we observed significant differences between SNc and SNr, with the latter having more IBA1+ cells than SNc (p value<0.005) (**Figure 4C**). Then, to address the complexity of microglia morphology, we analyzed three different parameters: total process length, number of branching points and number of segments. We first confirmed that even though all microglial cells in the mouse brain have a spindle-shaped-like morphology, cells in the cortex are far more complex than cerebellar cells. By applying this analysis to our regions of interest, we observed that microglia morphology in the striatum was similar to the corresponding cells in the cortex, whereas the lower microglia complexity detected in the cerebellum was similar to midbrain (**Figures 4D** **–** **G**). We did not detect significant morphological microglia sub-regional variation within midbrain and striatum (**Table S5**).

Taken together, our results confirm the spatial heterogeneity of microglia across different brain regions and further demonstrate organizational and morphological diversities between midbrain and striatum, thus supporting the transcriptional signatures identified at single-cell resolution suggesting that microglia functionality within these regions may be heterogeneous.

## Discussion

Neuroinflammation linked to chronic or abnormal microglia activation is supposed to contribute to the loss of dopaminergic neurons in PD (46–48). Microglia actively contribute to PD pathology by reacting to α-syn (49). However, it is still not clear if different microglial cell populations in the healthy brain might be more prone to aberrant activation, which might consequently induce and sustain neurodegeneration under threatening conditions. To address this question, transcriptomic differences of microglia across specific brain regions have been studied over the last years (17, 50). For example, various bulk approaches elucidated microglia features across cortex, cerebellum, hippocampus or the basal ganglia (21, 22, 51). These studies confirmed the diversity of microglia across these brain regions, with microglia in the hippocampus and cerebellum displaying a marked immune vigilant status when compared to cortex and basal ganglia. However, bulk analyses are not suitable to identify specific cellular subsets, since they merely represent the average of specific cellular programs. To overcome this difficulty, single-cell approaches aided to detect, for example, a higher microglia heterogeneity during development (23, 52). In the adult brain, heterogeneity has been primarily linked to specialized microglial cells displaying different predisposition to be activated (23, 50, 52–55). Here, we applied the Drop-seq method (30) to elucidate microglia heterogeneity within the nigrostriatal pathway, the main affected path in PD. First, we took advantage of this technique to decipher the cell taxonomy across midbrain and striatum, revealing the identity of all the main brain cells in line with other studies identifying similar cell types in the cortex, hippocampus or striatum (32, 56, 57). Intriguingly, we detected few cells originally included in the microglia cluster expressing oligodendrocytic markers that we discarded for further analysis. We are currently investigating the biological or technical relevance of these detected cells.

When focusing on microglia heterogeneity in our studied regions, we identified a special subset prevalently composed by cells of the midbrain. The corresponding upregulated genes were mainly related to immune and inflammatory response, thus indicating that this subset display an “immune alerted” phenotype. Notably, microglial activation and inflammation have been reported to contribute to neurodegeneration in *in vivo* models of PD by directly affecting the dopaminergic neurons (47, 58). In addition, midbrain-enriched immune alerted microglial cells were enriched for toll-like receptor (TLR) signaling pathways, which have been linked to neurodegeneration (59). Specifically, TLR4 is involved in microglia activation linked to PD and the lack of *Tlr4* in a PD-like model of MPTP resulted in a reduced microglia activation and a decreased neurodegeneration (60). Further, it has been shown that TLR4 is necessary for α-syn uptake by microglia and their subsequent activation (61). We also identified enrichment expression of antigen processing and presentation markers, including MHC-II signaling pathway. MHC-II is upregulated by microglia in post-mortem brains of PD patients (62) and *in vitro* models have shown its relation between microglia activation and further neurodegeneration (63), thus indicating that MHC-II may be a mediator directly involved in PD pathogenesis.

Experimentally, peripheral injection of lipopolysaccharide (LPS) in the mouse has been related to neurodegeneration (64) by contributing to the aggregation of different proteins such as α-syn (65). In addition, the consecutive injection of LPS aggravate the loss of dopaminergic neurons, which occurs *via* the activation of the microglial complement-phagosome pathway (47). Taken together, this might indicate that the immune alerted phenotype, mainly displayed by midbrain microglia and few striatal microglia, might turn detrimental in the long term or under specific cues, contributing to dopaminergic neuronal loss triggering PD-like pathogenesis. For example, aged microglia exhibit a primed state characterized by a hyper-reactive response towards threatening conditions. As a consequence, primed microglia release higher amounts of cytokines and chemokines that could turn neurotoxic, thus contributing to disease progression (66). Intriguingly, following LPS administration, midbrain microglia show an immunosuppressive response when compared to microglia from other brain regions, including the cortex, striatum or hippocampus, indicating that microglia in the midbrain display a dampened response towards an inflammatory insult, hence presenting a tolerogenic and not a primed phenotype (67). Further, the analysis of microglia phenotypes associated with chronic inflammation in a TNF transgenic mouse model recently revealed distinct signatures across different brain regions, including the cortex, striatum, hippocampus, thalamus and cerebellum. More specifically, microglial cells located within the cortex, striatum and thalamus were characterized by the overexpression of inflammatory genes, such as *Cxcl13*, *Ccl2*, *C3* and *C4b*, thus suggesting a more pronounced reactive state of microglia under persistent inflammation in these specific regions when compared to hippocampus and cerebellum (68). These results are in line with our observations since it has been previously shown that, at baseline, hippocampal and cerebellar microglia exist in a more immune-vigilant state (21). Hence, the intrinsic immune-alerted phenotype in the midbrain evidenced by our results as well as the previously described immune-vigilant state in the hippocampus and cerebellum seem to confer microglia the ability to react to an inflammatory stimulus at a lesser extent compared to the corresponding cells in other analyzed brain regions. In the midbrain, whether this putative reduced response might be detrimental for the underlying dopaminergic neuronal network as, for example, threatening conditions cannot be efficiently resolved, remains a matter of investigation. In addition, whether midbrain microglia will similarly respond to a PD-like insult, such as α-syn aggregation or neuronal loss, requires further analyses.

Concomitantly to transcriptional adaptations, morphological changes of microglia also underlie their activated state. Briefly, amoeboid microglia with shorter or thicker ramifications are linked to an activated state, whereas highly ramified phenotypes are associated to classical homeostatic microglia (69–71). In this context, microglia complexity has been described to vary depending on the localization of the cells across specific brain regions (72). Indeed, microglia from cortex, hippocampus and striatum are more complex than microglia from cerebellum (54, 73). In addition, microglia density decreases during the rise of dystrophic and degenerating cells in the aging mouse nigrostriatal pathway (74). In these perspectives, the development of computational and machine learning approaches recently enabled the identification of 9 microglia subsets based on 62 morphological features in the murine hippocampus (75). To complement our analyses, it will be critical to use these approaches to further unraveling clusters of cells across the analyzed brain regions.

Lastly, the recently described heterogeneity of neuronal populations in the nigrostriatal pathway supports our results on microglia diversity in the corresponding brain region. For example, seven subsets of dopaminergic neurons have been identified in the mouse brain, which are also present in humans (76, 77). These studies also highlight the importance of investigating cellular heterogeneity in the murine nigrostriatal pathway, since this region is mainly conserved across mouse and human species.

The combination of single-cell transcriptomics and *in situ* morphological approaches to study microglia phenotypes across the nigrostriatal pathway at baseline enabled us to detect a small subset of cells, mainly constituted by microglia in the midbrain, displaying an immune alerted phenotype, which might have implications in PD. Whether those immune alerted microglia are sustained by specific cues in the midbrain environment, such as mediators released by the highly active dopaminergic neuronal network, or they are ontogenetically different compared to other brain regions needs further investigations. Additional studies elucidating microglia immune phenotypes in relevant PD mouse models and patients, both in males and females, using single-cell transcriptional and imaging approaches, such as imaging mass cytometry, will be critical to understand how different subsets of cells might be beneficial or detrimental during the development and progression of the disease.

## Data Availability Statement

The datasets presented in this study can be found in online repositories. The names of the repository/repositories and accession number(s) can be found at: https://www.ncbi.nlm.nih.gov/geo/ GSE148393.

## Ethics Statement

The animal study was reviewed and approved by Animal Experiment Ethics Committee of the University of Luxembourg and the responsible Luxembourg government authorities (Ministry of Health, Ministry of Agriculture).

## Author Contributions

OUH, MB, MM, and AM designed project. AS set up single-cell analysis. OUH, TH, KG, YP-A, and RH performed experiments. OUH, DK, TH, KG, YP-A, RH, AS, MM, and AM analyzed experiments. MB, AS, MM, and AM supervised research. OUH and AM wrote the manuscript. All authors contributed to the article and approved the submitted version.

## Funding

OUH was supported by Art2Cure Foundation. DK was supported by the Luxembourg National Research Fund (FNR) through PRIDE17/12244779/PARK-QC. YP-A was supported by the FNR through PRIDE15/10675146/CANBIO and by the Fondation du Pélican de Mie et Pierre Hippert-Faber (Fondation de Luxembourg). AS and KG were supported by the FNR through the C14/BM/7975668/CaSCAD and INTER/DFG/17/11583046 projects as well as by the National Biomedical Computation Resource (NBCR) through the NIH P41 GM103426 grant from the National Institutes of Health. MM would like to thank the FNR for the support (PEARL P16/BM/11192868 grant). Rotary Club Luxembourg in the framework of its initiative “Espoir-en-tête” supported MB, AS, and AM. We acknowledge financial support by the Luxembourg Institute of Health and the Luxembourg Centre for Systems Biomedicine (MIGLISYS).

## Conflict of Interest

The authors declare that the research was conducted in the absence of any commercial or financial relationships that could be construed as a potential conflict of interest.

## Supplementary Material

Supplementary Figure 1 Cellular taxonomy across midbrain and striatum. **(A)** Heatmap showing clustering analysis featuring 15 most variable genes per cluster (FDR < 0.05). Color bar represents z-scores (from low z-score in blue to high z-score in red). **(B)** t-SNE representation of cell-type representative genes. Color bar represents z-scores (high z-score in red).

Supplementary Figure 2 Enrichment of microglial cells across midbrain and striatum. **(A)** Left panel: UMAP representation of microglial cells from the midbrain (in green) and striatum (in orange); right panel: UMAP representation showing four distinct subsets with few microglial cells (in purple) clustering independently. **(B)** Heatmap showing the corresponding microglial cells expressing oligodendrocytic markers (padj < 0.05).

Supplementary Figure 3 Immune phenotypes across specific brain regions. **(A)** Flow cytometry gating strategy. (i) Cells of interest were gated based on forward-scatter area (FSC-A) and side-scatter area (SSC-A). (ii) Doublets were excluded based on forward-scatter area (FSC-A) versus forward-scatter height (FSC-H). (iii) Hoechst was used to discriminate dead and live cells. (iv) CD45-FITC and CD11b-PERCP_cy5.5 antibodies were used to gate microglia. (v) CD83-PE antibody was used to quantify CD83 expression. (vi) CD45-FITC and CD11b-PERCP_cy5.5 antibodies were used to gate the myeloid compartment. (vii) CD206-APC antibody was used to quantify CD206 expression. **(B)** *t*-SNE and UMAP plots showing *Mrc1* expression across cell types (left) and microglia (right), respectively. Color bars represent z-scores (from low z-score in grey to high z-score in red). **(C)** Percentage of CD206+ cells within the CD11b+CD45+ population quantified by flow cytometry. Bars represent mean ± SEM (cortex in pale green; striatum in orange; midbrain in green). Unpaired Student t test (n = 3). **(D)** Expression levels of microglia homeostatic gene markers in microglia isolated from cortex (in pale green), cerebellum (in pink), striatum (in orange) and midbrain (in green) analyzed by qPCR. *Gapdh* was used as housekeeping gene. Bars represent the mean ± standard error of the mean (SEM) from three independent experiments. One-way ANOVA followed by a post-hoc Tukey’s test was used to infer statistical differences between groups (*p < 0.05).
