# Supplementary figures and images for: Single-Cell Transcriptomics and In Situ Morphological Analyses Reveal Microglia Heterogeneity Across the Nigrostriatal Pathway
Source: Front Immunol. 2021 Mar 29;12:639613. doi: 10.3389/fimmu.2021.639613 (PMC8039119; doi:10.3389/fimmu.2021.639613)

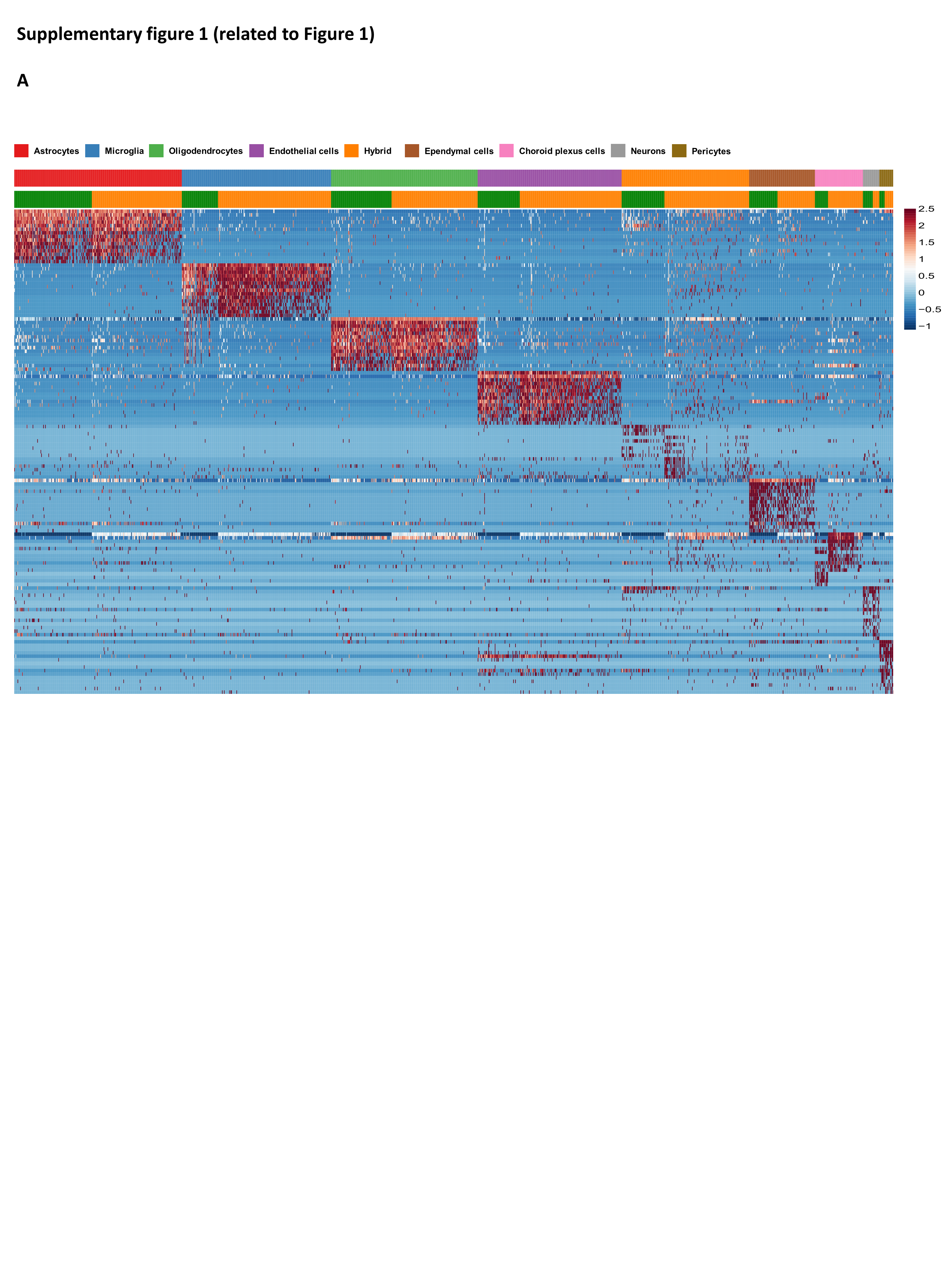

Supplement: Supplementary Figure 1 — Cellular taxonomy across midbrain and striatum. (A) Heatmap showing clustering analysis featuring 15 most variable genes per cluster (FDR < 0.05). Color bar represents z-scores (from low z-score in blue to high z-score in red). (B) t-SNE representation of cell-type representative genes. Color bar represents z-scores (high z-score in red). [file Presentation_1.zip › Supplementary Figure 1A.TIF]

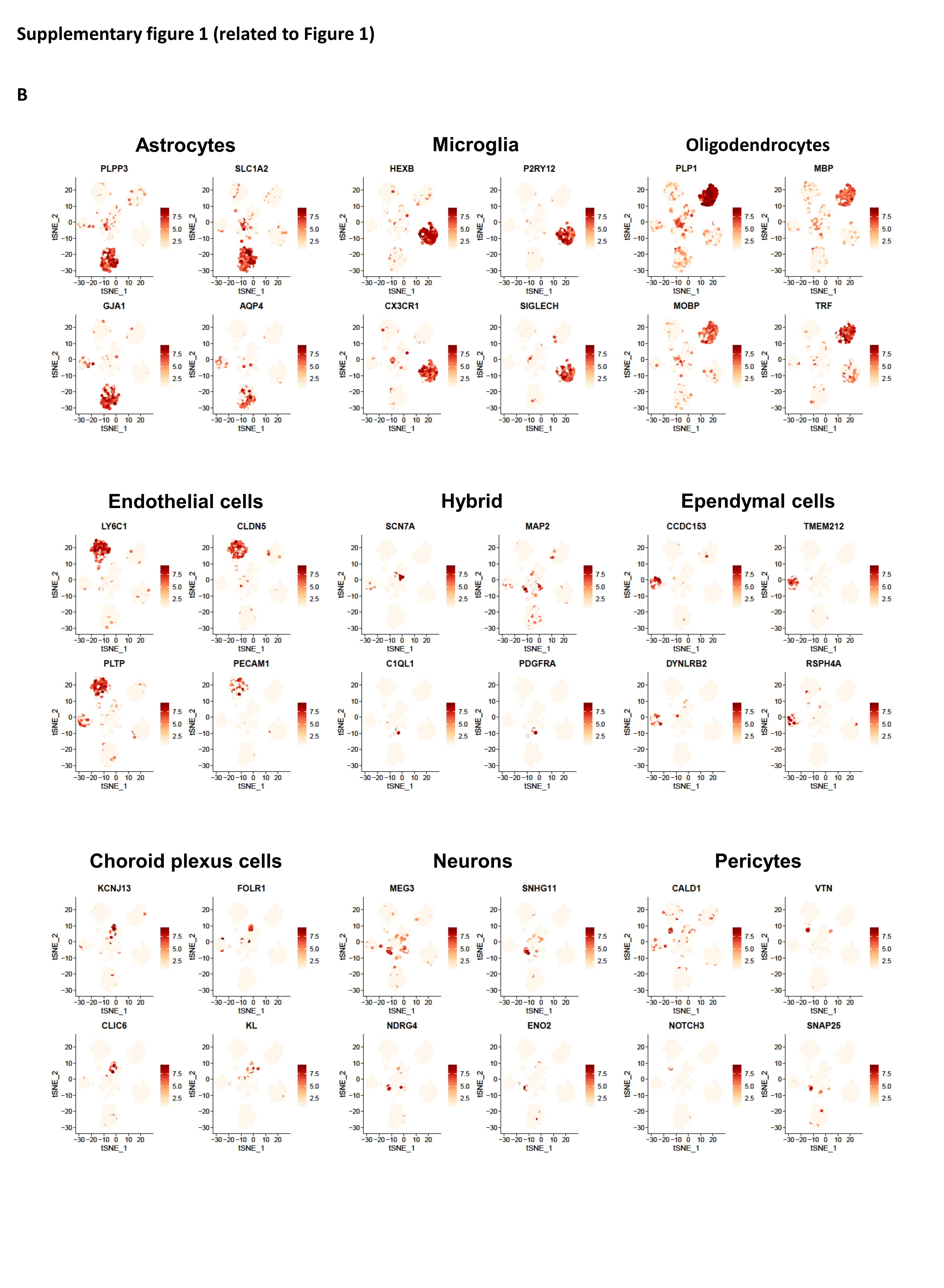

Supplement: Supplementary Figure 1 — Cellular taxonomy across midbrain and striatum. (A) Heatmap showing clustering analysis featuring 15 most variable genes per cluster (FDR < 0.05). Color bar represents z-scores (from low z-score in blue to high z-score in red). (B) t-SNE representation of cell-type representative genes. Color bar represents z-scores (high z-score in red). [file Presentation_1.zip › Supplementary Figure 1B.TIF]

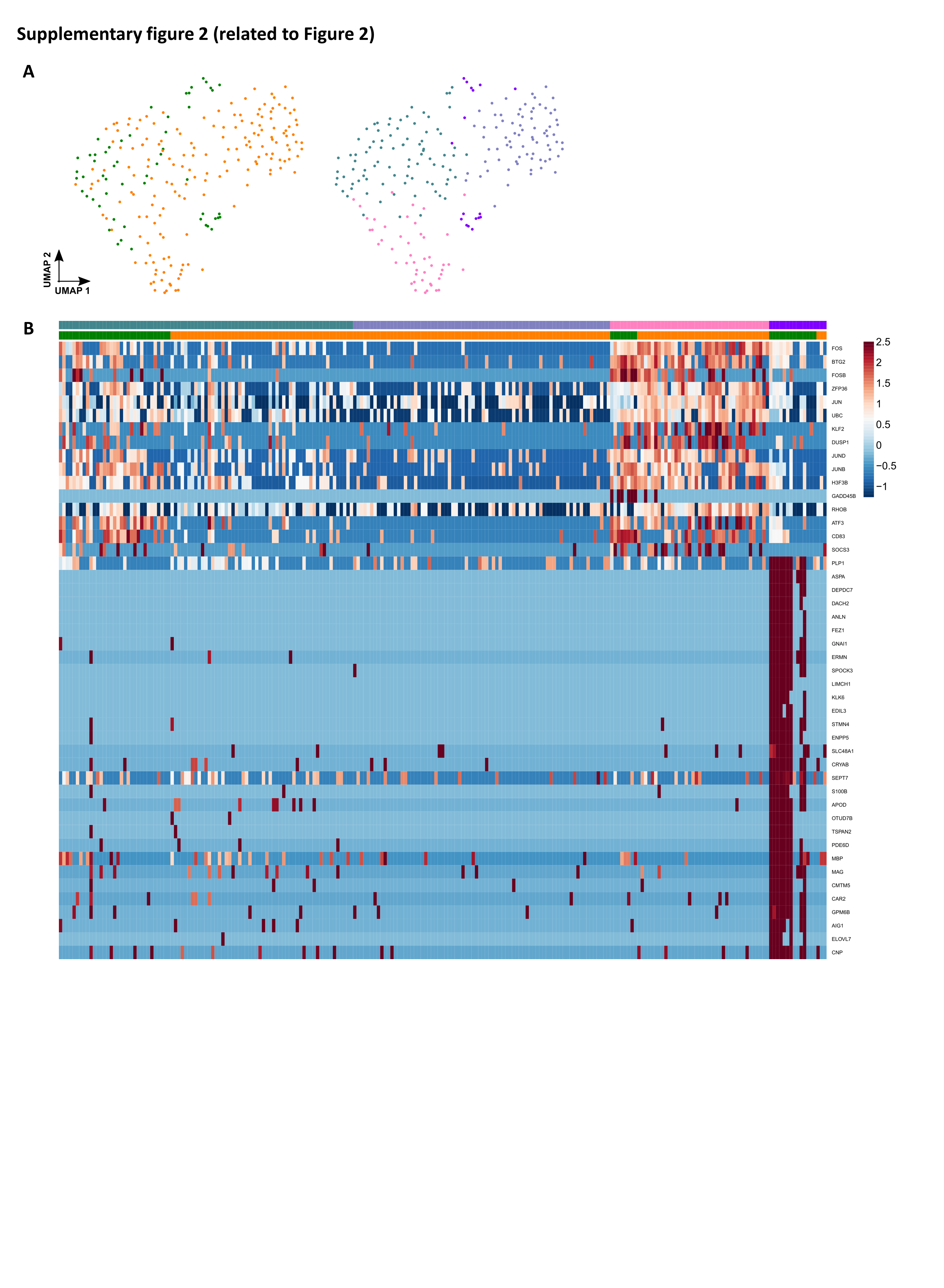

Supplement: Supplementary Figure 1 — Cellular taxonomy across midbrain and striatum. (A) Heatmap showing clustering analysis featuring 15 most variable genes per cluster (FDR < 0.05). Color bar represents z-scores (from low z-score in blue to high z-score in red). (B) t-SNE representation of cell-type representative genes. Color bar represents z-scores (high z-score in red). [file Presentation_1.zip › Supplementary Figure 2A.TIF]

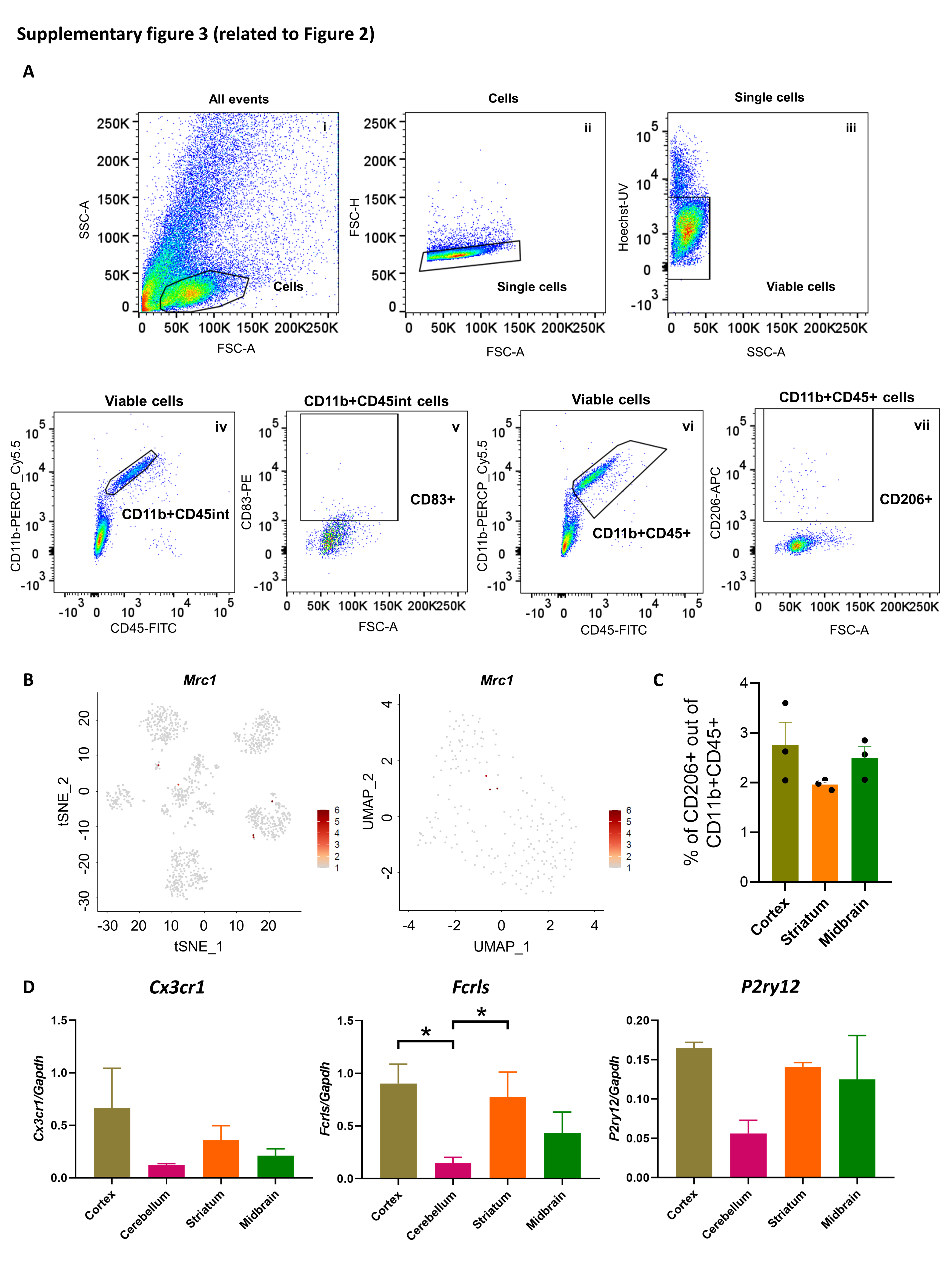

Supplement: Supplementary Figure 1 — Cellular taxonomy across midbrain and striatum. (A) Heatmap showing clustering analysis featuring 15 most variable genes per cluster (FDR < 0.05). Color bar represents z-scores (from low z-score in blue to high z-score in red). (B) t-SNE representation of cell-type representative genes. Color bar represents z-scores (high z-score in red). [file Presentation_1.zip › Supplementary Figure 2B.TIF]
