# Supplementary material for: Single-Cell Transcriptomics and In Situ Morphological Analyses Reveal Microglia Heterogeneity Across the Nigrostriatal Pathway
Source: Front Immunol. 2021 Mar 29;12:639613. doi: 10.3389/fimmu.2021.639613 (PMC8039119; doi:10.3389/fimmu.2021.639613)
Supplement: Supplementary file 2 [file Table_1.docx]

**Table S1. Top genes expressed by each cluster (padj value < 0.01; logFC > 0.5).**

| Gene symbol | padj value | LogFC | Cell type |
| --- | --- | --- | --- |
| *Agt* | 3.30E-93 | 2.99 | Astrocytes |
| *Slc1a2* | 2.82E-173 | 2.98 | Astrocytes |
| *Slc4a4* | 1.58E-121 | 2.94 | Astrocytes |
| *Ttyh1* | 2.10E-169 | 2.93 | Astrocytes |
| *Slc6a11* | 4.29E-114 | 2.91 | Astrocytes |
| *Plpp3* | 2.63E-174 | 2.90 | Astrocytes |
| *Pla2g7* | 2.33E-125 | 2.88 | Astrocytes |
| *Ntsr2* | 9.30E-134 | 2.81 | Astrocytes |
| *Slc1a3* | 2.25E-140 | 2.78 | Astrocytes |
| *Gja1* | 1.67E-123 | 2.76 | Astrocytes |
| *Ctss* | 1.92E-200 | 3.82 | Microglia |
| *C1qc* | 3.03E-192 | 3.61 | Microglia |
| *C1qb* | 3.11E-184 | 3.56 | Microglia |
| *Hexb* | 1.74E-203 | 3.53 | Microglia |
| *C1qa* | 5.28E-179 | 3.52 | Microglia |
| *P2ry12* | 1.31E-146 | 3.32 | Microglia |
| *Csf1r* | 2.63E-137 | 3.00 | Microglia |
| *Selplg* | 6.41E-119 | 2.89 | Microglia |
| *Siglech* | 2.15E-107 | 2.86 | Microglia |
| *Tyrobp* | 1.23E-115 | 2.85 | Microglia |
| *Plp1* | 2.44E-282 | 3.39 | Oligodendrocytes |
| *Cldn11* | 5.30E-155 | 3.13 | Oligodendrocytes |
| *Mag* | 9.71E-159 | 3.07 | Oligodendrocytes |
| *Ermn* | 5.74E-159 | 3.05 | Oligodendrocytes |
| *Cryab* | 7.50E-130 | 2.87 | Oligodendrocytes |
| *Mal* | 3.61E-130 | 2.75 | Oligodendrocytes |
| *Apod* | 8.55E-136 | 2.74 | Oligodendrocytes |
| *Car2* | 1.16E-132 | 2.66 | Oligodendrocytes |
| *Mog* | 3.26E-100 | 2.64 | Oligodendrocytes |
| *Ly6c1* | 1.15E-171 | 3.64 | Endothelial cells |
| *Cldn5* | 5.41E-150 | 3.37 | Endothelial cells |
| *Flt1* | 7.05E-150 | 3.37 | Endothelial cells |
| *Ly6a* | 8.16E-148 | 3.32 | Endothelial cells |
| *Itm2a* | 2.96E-115 | 3.05 | Endothelial cells |
| *Adgrf5* | 3.16E-134 | 2.92 | Endothelial cells |
| *Abcb1a* | 1.21E-111 | 2.82 | Endothelial cells |
| *Cxcl12* | 6.35E-92 | 2.81 | Endothelial cells |
| *Bsg* | 8.56E-159 | 2.71 | Endothelial cells |
| *Spock2* | 2.13E-100 | 2.66 | Endothelial cells |
| *Scn7a* | 1.05E-20 | 2.39 | Hybrid |
| *6330403k07rik* | 1.21E-29 | 2.23 | Hybrid |
| *Dlx1* | 4.44E-18 | 1.89 | Hybrid |
| *Dlx6os1* | 3.12E-16 | 1.81 | Hybrid |
| *Meis2* | 7.09E-11 | 1.76 | Hybrid |
| *Dlk1* | 2.51E-18 | 1.67 | Hybrid |
| *Sox11* | 6.04E-14 | 1.65 | Hybrid |
| *Mest* | 1.33E-17 | 1.65 | Hybrid |
| *Stmn2* | 1.42E-11 | 1.63 | Hybrid |
| *Vcan* | 1.48E-15 | 1.63 | Hybrid |
| *Ccdc153* | 3.67E-83 | 3.40 | Ependymal cells |
| *Tmem212* | 1.82E-74 | 3.20 | Ependymal cells |
| *Rarres2* | 3.60E-61 | 2.96 | Ependymal cells |
| *Rsph1* | 4.06E-53 | 2.78 | Ependymal cells |
| *Dynlrb2* | 1.86E-54 | 2.65 | Ependymal cells |
| *Rsph4a* | 6.62E-53 | 2.64 | Ependymal cells |
| *Enkur* | 5.65E-54 | 2.61 | Ependymal cells |
| *Fam183b* | 1.14E-49 | 2.54 | Ependymal cells |
| *Gm45396* | 1.44E-50 | 2.54 | Ependymal cells |
| *Cfap161* | 1.16E-45 | 2.50 | Ependymal cells |
| *Ttr* | 1.44E-99 | 3.47 | Choroid plexus cells |
| *Prg4* | 6.37E-07 | 3.21 | Choroid plexus cells |
| *1500015O10RIK* | 1.57E-45 | 2.80 | Choroid plexus cells |
| *Mgp* | 3.22E-07 | 2.54 | Choroid plexus cells |
| *Efemp1* | 4.55E-11 | 2.46 | Choroid plexus cells |
| *Enpp2* | 1.60E-49 | 2.40 | Choroid plexus cells |
| *Nov* | 4.02E-12 | 2.28 | Choroid plexus cells |
| *SLc4a10* | 3.03E-22 | 2.12 | Choroid plexus cells |
| *Sostdc1* | 1.12E-27 | 2.12 | Choroid plexus cells |
| *Prlr* | 7.42E-22 | 2.04 | Choroid plexus cells |
| *Meg3* | 1.25E-24 | 4.07 | Neurons and neural stem cells |
| *Snhg11* | 5.91E-19 | 3.90 | Neurons and neural stem cells |
| *Snap25* | 6.65E-10 | 2.90 | Neurons and neural stem cells |
| *9330162g02rik* | 1.58E-09 | 2.71 | Neurons and neural stem cells |
| *Ndrg4* | 1.15E-12 | 2.69 | Neurons and neural stem cells |
| *Ryr2* | 3.39E-06 | 2.58 | Neurons and neural stem cells |
| *Dnm1* | 1.84E-09 | 2.44 | Neurons and neural stem cells |
| *Camk2a* | 2.41E-06 | 2.44 | Neurons and neural stem cells |
| *Tac1* | 4.13E-04 | 2.43 | Neurons and neural stem cells |
| *Cplx2* | 4.82E-06 | 2.39 | Neurons and neural stem cells |
| *Vtn* | 2.65E-21 | 4.37 | Pericytes |
| *Rgs5* | 3.77E-21 | 3.90 | Pericytes |
| *Myh11* | 1.41E-06 | 3.36 | Pericytes |
| *Cald1* | 9.46E-25 | 3.32 | Pericytes |
| *Acta2* | 4.36E-07 | 3.26 | Pericytes |
| *Myl9* | 1.01E-22 | 3.26 | Pericytes |
| *Atp13a5* | 2.95E-10 | 2.68 | Pericytes |
| *Crip1* | 7.92E-08 | 2.64 | Pericytes |
| *Gucy1b3* | 5.97E-10 | 2.50 | Pericytes |
| *TPM1* | 2.85E-09 | 2.47 | Pericytes |
