# Supplementary material for: Single-Cell Transcriptomics and In Situ Morphological Analyses Reveal Microglia Heterogeneity Across the Nigrostriatal Pathway
Source: Front Immunol. 2021 Mar 29;12:639613. doi: 10.3389/fimmu.2021.639613 (PMC8039119; doi:10.3389/fimmu.2021.639613)
Supplement: Supplementary file 3 [file Table_2.docx]

**Table S2. Differentially expressed genes across microglia subsets (q value < 0.05).**

| Gene symbol | q value | LogFC | Subset |
| --- | --- | --- | --- |
| *Atf3* | 7.95E-31 | 1.40 | Immune alerted |
| *Egr1* | 1.66E-15 | 1.17 | Immune alerted |
| *Cd74* | 2.27E-09 | 1.15 | Immune alerted |
| *Junb* | 3.35E-15 | 1.11 | Immune alerted |
| *Cd83* | 3.94E-12 | 1.01 | Immune alerted |
| *Nfkbiz* | 1.48E-15 | 0.96 | Immune alerted |
| *Ccl4* | 4.96E-14 | 0.95 | Immune alerted |
| *H2-K1* | 1.35E-06 | 0.81 | Immune alerted |
| *Klf2* | 6.89E-07 | 0.79 | Immune alerted |
| *Fosb* | 1.72E-08 | 0.73 | Immune alerted |
| *Adamts1* | 3.90E-10 | 0.72 | Immune alerted |
| *H3f3b* | 1.74E-05 | 0.72 | Immune alerted |
| *Il1b* | 1.17E-09 | 0.72 | Immune alerted |
| *Nfkbia* | 1.12E-05 | 0.69 | Immune alerted |
| *Rel* | 3.01E-07 | 0.66 | Immune alerted |
| *Gpx1* | 1.71E-03 | 0.66 | Immune alerted |
| *Fos* | 3.09E-04 | 0.64 | Immune alerted |
| *Fth1* | 5.21E-03 | 0.63 | Immune alerted |
| *Ier5* | 2.04E-03 | 0.60 | Immune alerted |
| *Btg2* | 8.02E-04 | 0.60 | Immune alerted |
| *Ier2* | 6.11E-05 | 0.59 | Immune alerted |
| *Cd14* | 4.20E-02 | 0.58 | Immune alerted |
| *Jund* | 3.94E-02 | 0.56 | Immune alerted |
| *Evi2a* | 2.08E-02 | 0.53 | Immune alerted |
| *Fcrls* | 7.65E-05 | 0.52 | Intermediate 1 |
| *Il1a* | 7.92E-05 | 0.50 | Immune alerted |
| *Dusp1* | 1.30E-02 | 0.47 | Immune alerted |
| *Klf6* | 3.02E-03 | 0.46 | Immune alerted |
| *Gm10269* | 7.05E-04 | 0.44 | Immune alerted |
| *H2-Ab1* | 2.47E-03 | 0.44 | Immune alerted |
| *P2ry12* | 4.37E-03 | 0.43 | Intermediate 1 |
| *Icam1* | 2.40E-03 | 0.43 | Immune alerted |
| *Ttr* | 1.05E-08 | 0.43 | Intermediate 1 |
| *Mcl1* | 1.16E-02 | 0.43 | Immune alerted |
| *Mir692-1* | 1.55E-02 | 0.42 | Immune alerted |
| *Gm12346* | 2.26E-02 | 0.41 | Immune alerted |
| *Socs3* | 2.26E-02 | 0.41 | Immune alerted |
| *Gm17087* | 1.16E-02 | 0.41 | Immune alerted |
| *Ubc* | 1.59E-03 | 0.41 | Immune alerted |
| *Fubp1* | 1.09E-02 | 0.40 | Homeostatic |
| *H2-Aa* | 4.33E-03 | 0.38 | Immune alerted |
| *Gpr84* | 2.86E-02 | 0.38 | Immune alerted |
| *Pcf11* | 6.62E-03 | 0.35 | Immune alerted |
| *Gm7290* | 4.37E-02 | 0.35 | Immune alerted |
| *Sdc4* | 4.09E-02 | 0.34 | Immune alerted |
| *Itm2b* | 1.07E-02 | 0.34 | Intermediate 1 |
| *Jun* | 2.94E-03 | 0.33 | Immune alerted |
| *Csf1* | 2.27E-03 | 0.33 | Immune alerted |
| *Casp4* | 1.09E-02 | 0.33 | Immune alerted |
| *Hspa1a* | 1.16E-02 | 0.32 | Homeostatic |
| *Hexb* | 2.61E-02 | 0.31 | Homeostatic |
| *C1qa* | 3.30E-04 | 0.31 | Intermediate 2 |
| *Zcchc17* | 3.43E-02 | 0.30 | Immune alerted |
| *Lgmn* | 3.40E-03 | 0.28 | Intermediate 1 |
| *Rrad* | 1.16E-02 | 0.28 | Immune alerted |
| *Cx3cr1* | 1.83E-02 | 0.27 | Homeostatic |
| *Rnf216* | 4.37E-02 | 0.27 | Homeostatic |
| *Gm15536* | 2.07E-02 | 0.26 | Immune alerted |
| *Snapc1* | 2.62E-03 | 0.26 | Immune alerted |
| *Gm6159* | 7.35E-03 | 0.25 | Immune alerted |
| *Rbm8a* | 2.08E-02 | 0.25 | Immune alerted |
| *Gadd45b* | 4.72E-02 | 0.25 | Immune alerted |
| *Chd4* | 6.62E-03 | 0.23 | Intermediate 1 |
| *Sirt2* | 2.70E-02 | 0.23 | Immune alerted |
| *Cryl1* | 4.25E-02 | 0.22 | Immune alerted |
| *Cdkn1a* | 4.09E-02 | 0.22 | Immune alerted |
| *Lyz1* | 2.55E-02 | 0.22 | Immune alerted |
| *Tob2* | 4.66E-02 | 0.21 | Immune alerted |
| *Wdr26* | 4.20E-02 | 0.21 | Immune alerted |
| *Cggbp1* | 4.72E-02 | 0.21 | Immune alerted |
| *Sertad1* | 3.30E-02 | 0.20 | Immune alerted |
| *Tbca* | 4.72E-02 | 0.20 | Immune alerted |
| *Gm14747* | 4.03E-02 | 0.20 | Immune alerted |
| *Vps29* | 4.47E-02 | 0.19 | Intermediate 1 |
| *Dpy30* | 4.66E-02 | 0.18 | Immune alerted |
| *G3bp2* | 3.39E-02 | 0.17 | Intermediate 1 |
| *Gm5292* | 3.03E-02 | 0.16 | Immune alerted |
| *Atp5a1* | 3.78E-02 | 0.13 | Immune alerted |
