# Supplementary material for: Single-Cell Transcriptomics and In Situ Morphological Analyses Reveal Microglia Heterogeneity Across the Nigrostriatal Pathway
Source: Front Immunol. 2021 Mar 29;12:639613. doi: 10.3389/fimmu.2021.639613 (PMC8039119; doi:10.3389/fimmu.2021.639613)
Supplement: Supplementary file 4 [file Table_3.docx]

**Table S3. Biological processes and KEGG pathways identified by DAVID (p value < 0.05).**

| Biological Processes | p value |
| --- | --- |
| Cellular response to hormone stimulus | 7.01E-07 |
| Response to camp | 1.06E-06 |
| Positive regulation of transcription from RNA polymerase II promoter | 1.79E-06 |
| Response to cytokine | 1.07E-05 |
| Cellular response to calcium ion | 3.90E-05 |
| Regulation of cell cycle | 5.12E-05 |
| Immune response | 5.33E-05 |
| Negative regulation of cell proliferation | 6.75E-05 |
| Response to mechanical stimulus | 7.72E-05 |
| Transcription from RNA polymerase II promoter | 1.38E-04 |
| Inflammatory response | 2.29E-04 |
| Negative regulation of transcription from RNA polymerase II promoter | 2.56E-04 |
| Immune system process | 4.38E-04 |
| Ectopic germ cell programmed cell death | 4.51E-04 |
| Extrinsic apoptotic signaling pathway in absence of ligand | 4.60E-04 |
| Regulation of transcription from RNA polymerase II promoter | 5.43E-04 |
| Positive regulation of ERK1 and ERK2 cascade | 5.74E-04 |
| Response to lipopolysaccharide | 7.09E-04 |
| Transcription, DNA-templated | 8.27E-04 |
| Regulation of transcription, DNA-templated | 8.51E-04 |
| Negative regulation of apoptotic process | 9.21E-04 |
| Antigen processing and presentation | 9.65E-04 |
| Skeletal muscle cell differentiation | 1.02E-03 |
| Antigen processing and presentation of exogenous peptide antigen via MHC class II | 1.13E-03 |
| Response to drug | 1.35E-03 |
| Response to muscle stretch | 1.67E-03 |
| Cytokine-mediated signaling pathway | 1.89E-03 |
| Response to corticosterone | 2.56E-03 |
| Response to light stimulus | 3.62E-03 |
| Response to toxic substance | 3.67E-03 |
| Positive regulation of transcription, DNA-templated | 4.52E-03 |
| Response to radiation | 5.19E-03 |
| Lipopolysaccharide-mediated signaling pathway | 5.53E-03 |
| Negative regulation of extrinsic apoptotic signaling pathway in absence of ligand | 6.25E-03 |
| Protein kinase B signaling | 8.23E-03 |
| Negative regulation of T cell proliferation | 9.09E-03 |
| Negative regulation of cell growth | 1.03E-02 |
| Positive regulation of nitric oxide biosynthetic process | 1.14E-02 |
| Positive regulation of cell division | 1.14E-02 |
| Positive regulation of cell differentiation | 1.19E-02 |
| Positive regulation of cell death | 1.29E-02 |
| Positive regulation of gene expression | 1.43E-02 |
| Antigen processing and presentation of peptide antigen | 1.43E-02 |
| Fever generation | 1.78E-02 |
| Response to hydrogen peroxide | 1.85E-02 |
| Positive regulation of protein kinase activity | 2.22E-02 |
| Positive regulation of fibroblast proliferation | 2.29E-02 |
| Positive regulation of monocyte differentiation | 2.49E-02 |
| Positive regulation of neuron apoptotic process | 2.56E-02 |
| Positive regulation of JNK cascade | 2.63E-02 |
| Cellular response to organic cyclic compound | 2.98E-02 |
| Positive regulation of monocyte chemotactic protein-1 production | 3.19E-02 |
| Antigen processing and presentation of peptide or polysaccharide antigen via MHC class II | 3.19E-02 |
| Positive regulation of prostaglandin secretion | 3.19E-02 |
| Positive regulation of chemokine biosynthetic process | 3.19E-02 |
| Response to organic cyclic compound | 3.36E-02 |
| Cellular response to interleukin-1 | 3.36E-02 |
| Regulation of apoptotic process | 3.49E-02 |
| Regulation of cell death | 3.54E-02 |
| Toll-like receptor 4 signaling pathway | 3.88E-02 |
| Positive regulation of interleukin-2 biosynthetic process | 4.23E-02 |
| Negative regulation by host of viral transcription | 4.23E-02 |
| Cell proliferation | 4.48E-02 |
| Negative regulation of neural precursor cell proliferation | 4.92E-02 |
| Interleukin-1-mediated signaling pathway | 4.92E-02 |
| Mitotic cell cycle arrest | 4.92E-02 |
| KEGG pathways |  |
| Osteoclast differentiation | 3.44E-08 |
| Rheumatoid arthritis | 3.95E-07 |
| Leishmaniasis | 1.65E-06 |
| TNF signaling pathway | 2.76E-06 |
| Influenza A | 5.42E-06 |
| Herpes simplex infection | 2.28E-05 |
| HTLV-I infection | 2.46E-05 |
| Pertussis | 6.51E-05 |
| Salmonella infection | 8.39E-05 |
| MAPK signaling pathway | 8.70E-05 |
| Antigen processing and presentation | 1.07E-04 |
| Graft-versus-host disease | 2.26E-04 |
| Toll-like receptor signaling pathway | 2.85E-04 |
| Inflammatory bowel disease (IBD) | 3.68E-04 |
| Type I diabetes mellitus | 4.45E-04 |
| Prion diseases | 9.03E-04 |
| NF-kappa B signaling pathway | 2.38E-03 |
| Chagas disease (American trypanosomiasis) | 2.97E-03 |
| Staphylococcus aureus infection | 3.03E-03 |
| Tuberculosis | 3.45E-03 |
| Legionellosis | 4.39E-03 |
| Epstein-Barr virus infection | 7.95E-03 |
| Viral carcinogenesis | 1.08E-02 |
| Viral myocarditis | 1.09E-02 |
| Hematopoietic cell lineage | 1.32E-02 |
| Cell adhesion molecules (CAMs) | 1.45E-02 |
| Transcriptional misregulation in cancer | 1.54E-02 |
| Toxoplasmosis | 2.32E-02 |
| Allograft rejection | 4.22E-02 |
| Measles | 4.48E-02 |
