# Supplementary material for: Single-Cell Transcriptomics and In Situ Morphological Analyses Reveal Microglia Heterogeneity Across the Nigrostriatal Pathway
Source: Front Immunol. 2021 Mar 29;12:639613. doi: 10.3389/fimmu.2021.639613 (PMC8039119; doi:10.3389/fimmu.2021.639613)
Supplement: Supplementary file 5 [file Table_4.docx]

**Table S4. Differentially expressed genes in astrocytes, oligodendrocytes and ependymal cells between midbrain and striatum (LogFC > 1; adj p value < 0.05).**

| **Gene symbol** | **LogFC** | **Adj p value** | **Cell type** | **Brain region** |
| --- | --- | --- | --- | --- |
| ***Vwa1*** | 1.53 | 3.52e-07 | Astrocyte | Midbrain |
| ***Xpr1*** | 1.25 | 1.03e-05 | Astrocyte | Midbrain |
| ***H2-T3*** | 1.16 | 0.011 | Astrocyte | Midbrain |
| ***Foxb1*** | 1.21 | 0.015 | Astrocyte | Midbrain |
| ***Ttr*** | 4.21 | 1.76e-39 | Astrocyte | Striatum |
| ***Mfge8*** | 1.29 | 5.21e-6 | Astrocyte | Striatum |
| ***Tspan7*** | 1.09 | 0.004 | Astrocyte | Striatum |
| ***Atp1b1*** | 1.54 | 0.040 | Astrocyte | Striatum |
| ***Eif4h*** | 1.07 | 0.042 | Oligodendrocyte | Midbrain |
| ***Fos*** | 1.73 | 0.042 | Oligodendrocyte | Midbrain |
| ***Ttr*** | 3.78 | 1.01e-28 | Oligodendrocyte | Striatum |
| ***Ptma*** | 1.34 | 0.004 | Oligodendrocyte | Striatum |
| ***Car9*** | 2.48 | 6.7e-5 | Ependymal cell | Midbrain |
| ***Fam81b*** | 2.41 | 0.00018 | Ependymal cell | Midbrain |
| ***Atp5f1*** | 1.91 | 0.001 | Ependymal cell | Midbrain |
| ***Sparcl1*** | 1.03 | 0.004 | Ependymal cell | Midbrain |
| ***Cfap36*** | 1.23 | 0.020 | Ependymal cell | Midbrain |
| ***Fos*** | 2.29 | 0.021 | Ependymal cell | Midbrain |
| ***Ttr*** | 4.12 | 6.09e-12 | Ependymal cell | Striatum |
