# Supplementary material for: Single-Cell Transcriptomics and In Situ Morphological Analyses Reveal Microglia Heterogeneity Across the Nigrostriatal Pathway
Source: Front Immunol. 2021 Mar 29;12:639613. doi: 10.3389/fimmu.2021.639613 (PMC8039119; doi:10.3389/fimmu.2021.639613)
Supplement: Supplementary file 6 [file Table_5.docx]

**Table S5. Statistical values for microglia density and morphological analyses across brain regions.**

**A. Microglial cell density**

| **Tukey's multiple comparisons test** | **Significant** | **Summary** | **Adjusted P Value** |  |
| --- | --- | --- | --- | --- |
| CTX vs. CRB | Yes | *** | 0.0006 |  |
| CTX vs. CP | No | ns | 0.4003 |  |
| CTX vs. NA | No | ns | 0.2920 |  |
| CTX vs. SNr | Yes | ** | 0.0015 |  |
| CTX vs. SNc | Yes | * | 0.0240 |  |
| CTX vs. VTA | No | ns | 0.2521 |  |
| CRB vs. CP | Yes | **** | <0.0001 |  |
| CRB vs. NA | Yes | **** | <0.0001 |  |
| CRB vs. SNr | Yes | **** | <0.0001 |  |
| CRB vs. SNc | No | ns | 0.6766 |  |
| CRB vs. VTA | No | ns | 0.1175 |  |
| CP vs. NA | No | ns | >0.9999 |  |
| CP vs. SNr | No | ns | 0.1319 |  |
| CP vs. SNc | Yes | *** | 0.0002 |  |
| CP vs. VTA | Yes | ** | 0.0034 |  |
| NA vs. SNr | No | ns | 0.1946 |  |
| NA vs. SNc | Yes | *** | 0.0001 |  |
| NA vs. VTA | Yes | ** | 0.0021 |  |
| SNr vs. SNc | Yes | **** | <0.0001 |  |
| SNr vs. VTA | Yes | **** | <0.0001 |  |
| SNc vs. VTA | No | ns | 0.8829 |  |

**B. Dendrite total length**

| **Tukey's multiple comparisons test** | **Significant** | **Summary** | **Adjusted P Value** |
| --- | --- | --- | --- |
| CTX vs. CRB | Yes | *** | 0.0002 |
| CTX vs. CP | Yes | * | 0.0267 |
| CTX vs. NA | No | ns | 0.8263 |
| CTX vs. SNr | No | ns | 0.1072 |
| CTX vs. SNc | Yes | ** | 0.008 |
| CTX vs. VTA | Yes | *** | 0.0003 |
| CRB vs. CP | Yes | **** | <0.0001 |
| CRB vs. NA | Yes | **** | <0.0001 |
| CRB vs. SNr | No | ns | 0.4498 |
| CRB vs. SNc | No | ns | 0.9492 |
| CRB vs. VTA | No | ns | >0.9999 |
| CP vs. NA | No | ns | 0.4815 |
| CP vs. SNr | Yes | **** | <0.0001 |
| CP vs. SNc | Yes | **** | <0.0001 |
| CP vs. VTA | Yes | **** | <0.0001 |
| NA vs. SNr | Yes | ** | 0.0021 |
| NA vs. SNc | Yes | **** | <0.0001 |
| NA vs. VTA | Yes | **** | <0.0001 |
| SNr vs. SNc | No | ns | 0.9642 |
| SNr vs. VTA | No | ns | 0.5001 |
| SNc vs. VTA | No | ns | 0.9654 |

**C. Number of branching points**

| **Dunn's multiple comparisons test** | **Significant?** | **Summary** | **Adjusted P Value** |
| --- | --- | --- | --- |
| CTX vs. CRB | Yes | ** | 0.0089 |
| CTX vs. CP | No | ns | >0.9999 |
| CTX vs. NA | No | ns | >0.9999 |
| CTX vs. SNr | No | ns | >0.9999 |
| CTX vs. SNc | No | ns | 0.1353 |
| CTX vs. VTA | Yes | * | 0.0156 |
| CRB vs. CP | Yes | **** | <0.0001 |
| CRB vs. NA | Yes | *** | 0.0005 |
| CRB vs. SNr | No | ns | >0.9999 |
| CRB vs. SNc | No | ns | >0.9999 |
| CRB vs. VTA | No | ns | >0.9999 |
| CP vs. NA | No | ns | >0.9999 |
| CP vs. SNr | Yes | * | 0.0118 |
| CP vs. SNc | Yes | *** | 0.0003 |
| CP vs. VTA | Yes | **** | <0.0001 |
| NA vs. SNr | No | ns | 0.262 |
| NA vs. SNc | Yes | * | 0.0136 |
| NA vs. VTA | Yes | ** | 0.001 |
| SNr vs. SNc | No | ns | >0.9999 |
| SNr vs. VTA | No | ns | >0.9999 |
| SNc vs. VTA | No | ns | >0.9999 |

**D. Number of segments**

| **Dunn's multiple comparisons test** | **Significant?** | **Summary** | **Adjusted P Value** |  |
| --- | --- | --- | --- | --- |
| CTX vs. CRB | Yes | ** | 0.0076 |  |
| CTX vs. CP | No | ns | >0.9999 |  |
| CTX vs. NA | No | ns | >0.9999 |  |
| CTX vs. SNr | No | ns | >0.9999 |  |
| CTX vs. SNc | No | ns | 0.1133 |  |
| CTX vs. VTA | Yes | * | 0.0122 |  |
| CRB vs. CP | Yes | **** | <0.0001 |  |
| CRB vs. NA | Yes | *** | 0.0005 |  |
| CRB vs. SNr | No | ns | >0.9999 |  |
| CRB vs. SNc | No | ns | >0.9999 |  |
| CRB vs. VTA | No | ns | >0.9999 |  |
| CP vs. NA | No | ns | >0.9999 |  |
| CP vs. SNr | Yes | * | 0.0112 |  |
| CP vs. SNc | Yes | *** | 0.0003 |  |
| CP vs. VTA | Yes | **** | <0.0001 |  |
| NA vs. SNr | No | ns | 0.2501 |  |
| NA vs. SNc | Yes | * | 0.013 |  |
| NA vs. VTA | Yes | *** | 0.0009 |  |
| SNr vs. SNc | No | ns | >0.9999 |  |
| SNr vs. VTA | No | ns | >0.9999 |  |
| SNc vs. VTA | No | ns | >0.9999 |  |
